# A study on the role of Tai Chi training in improving type 2 diabetes mellitus

**DOI:** 10.3389/fpubh.2026.1730335

**Published:** 2026-02-06

**Authors:** Qiang Yang, Lincheng Li, Mingcai Sun, Hongcheng Luo, Chunyu Zhuang

**Affiliations:** 1 Physical Education Institution, Xichang University, Xichang, China; 2 Nursing Department, Haikou Maternal and Child Health Hospital, Haikou, China

**Keywords:** blood glucose homeostasis, gut microbiota, inflammatory factors, intestinal mucosal barrier function, Tai Chi training, type 2 diabetes mellitus

## Abstract

**Objective:**

To explore the role of Tai Chi training in improving Type 2 Diabetes Mellitus (T2DM) based on gut microbiota, serum inflammatory factors, and intestinal mucosal barrier function.

**Methods:**

Thirty-six patients with T2DM underwent 6 months of Tai Chi training. Body composition, biochemical indicators (fasting blood glucose, glycated hemoglobin, etc.), serum inflammatory factors (Tumor Necrosis Factor-α (TNF-α), Interleukin-6 (IL-6), etc.), and gut microbiota (16S rRNA sequencing) were measured.

**Results:**

After 6 months of Tai Chi training, significant reductions were observed in body weight, BMI, waist circumference, and body fat percentage (*p* < 0.05), while lean body mass increased significantly (*p* < 0.05). Fasting blood glucose, glycated hemoglobin, insulin resistance index (HOMA-IR), and total cholesterol levels decreased significantly (*p* < 0.01). C-reactive protein (CRP), TNF-α, IL-6, IL-1β, and IL-8 levels decreased significantly (*p* < 0.01), while the anti-inflammatory factor IL-10 increased significantly (*p* < 0.01). The Chao1 and Shannon indices increased significantly (*p* < 0.05). The abundance of beneficial bacteria increased significantly, while the abundance of harmful bacteria decreased significantly (*p* < 0.01). Markers of intestinal mucosal barrier function, including D-lactate and zonulin, decreased significantly (*p* < 0.01), while the level of milk fat globule-EGF factor 8 (MFG-E8) increased significantly (*p* < 0.01).

**Conclusion:**

Tai Chi training can improve blood glucose homeostasis, gut microbiota richness and diversity, intestinal mucosal barrier function, and systemic inflammatory status in T2DM patients. Tai Chi training may be an important approach for personalized treatment of T2DM.

## Introduction

Type 2 diabetes mellitus (T2DM), as the most common metabolic disease at present, has seen a continuous increase in the number of patients over the past 50 years, and has shown a trend of spreading from Western countries to Asia, Africa, and other Western Pacific countries. In addition, according to existing models, it is estimated that by 2045, nearly 700 million people worldwide will be affected by this disease (1). The prevalence of diabetes among Chinese adults has reached 11.2%, with T2DM accounting for more than 90% (2). Insulin resistance is considered the main cause of the development of T2DM. As the disease progresses, T2DM can cause a variety of serious complications, such as cardiovascular disease, retinopathy, neuropathy, and nephropathy, which seriously affect the quality of life and prognosis of patients (3). Today, the disease has become one of the global public health issues, seriously endangering human health and causing huge social burdens and economic losses. Researchers are actively studying its underlying pathophysiological mechanisms in order to propose innovative treatment strategies. Clinical evidence indicates that both genetic and environmental factors can cause and exacerbate T2DM (4). In addition, studies have shown that adipose tissue and macrophages in T2DM patients release large amounts of pro-inflammatory factors, such as tumor mecrosis factor-α (TNF-α), interleukin-6 (IL-6), and monocyte chemoattractant protein-1 (MCP-1). These factors can inhibit the insulin signaling pathway and worsen insulin resistance. They can also activate catabolic pathways, thereby leading to T2DM complications (5).

An increasing amount of evidence shows that the gut microbiota is associated with the development of T2DM through multiple pathways. The gut microbiota can affect the integrity of the intestinal epithelial barrier, mediate insulin resistance, and regulate mitochondrial function. These microbiota can also regulate local or systemic immunity and inflammation, which contributes to the development of T2DM. In addition, a variety of gut microbial metabolites, such as short-chain fatty acids, bile acids, and tryptophan-derived metabolites, have been reported to be closely related to the pathogenesis of T2DM (6). As it is well known, exercise plays an important role in preventing diseases and controlling blood glucose as well as diabetes-related organ complications. In fact, aerobic exercise is often used to prevent and control diabetes (7). Studies have shown that aerobic training increases skeletal muscle insulin responsiveness by increasing the expression and/or activity of enzymes involved in cellular glucose utilization (8). Moreover, physical activity has a positive impact on blood lipids, blood pressure, and body weight, as it can reduce cardiovascular risk and mortality in both healthy and affected populations (9). Recent studies have shown that regular physical activity can also affect the composition of the microbiota and immune responses (10). Tai Chi training is one of the traditional Chinese fitness exercises and an excellent traditional Chinese sports item. Studies have shown that Tai Chi training has a significant improvement effect on blood glucose and blood lipid levels in T2DM patients (11). According to other studies, Tai Chi training can also have a positive effect on the gut microbiota of the elderly, mainly manifested in the fact that long-term practice of Tai Chi training can increase the number of beneficial bacteria (*Bifidobacterium, Lactobacillus*) in the intestines of the elderly and reduce the number of harmful bacteria (*Enterobacteriaceae, Fusobacterium*, and *Enterococcus*) (12). This has confirmed the fitness effect of Tai Chi training from a microbiological perspective. However, the impact of Tai Chi training on the gut microbiota of T2DM patients and its correlation with IR remains to be further studied. This study aims to explore the effects of long-term Tai Chi training on blood glucose homeostasis, systemic inflammatory status, gut microbiota composition, and intestinal mucosal barrier function in T2DM patients. It provides a theoretical basis for the prevention and treatment of metabolic diseases through exercise, and lays a theoretical foundation for promoting the positive role of Tai Chi training, an excellent traditional Chinese sports item, in Healthy China and the building of a community with a shared future for mankind. Based on the aforementioned background, this study formulated the following hypotheses: (1) Long-term Tai Chi training can effectively improve glycemic homeostasis and significantly alleviate insulin resistance in T2DM patients; (2) Tai Chi training can optimize the gut microbiota structure in T2DM patients by increasing the abundance of beneficial bacteria and inhibiting the growth of potential pathogens; (3) Tai Chi training can reduce systemic pro-inflammatory factor levels and enhance intestinal mucosal barrier function; (4) The alterations in gut microbiota composition and the strengthening of barrier function are significantly correlated with the improvement of clinical metabolic parameters and inflammatory status.

## Research subjects and methods

### Ethical statement and study design

This study employed a self-controlled pre-and-post intervention design. The study protocol was reviewed and approved by the Ethics Committee of Xi Chang University (Approval No. LLPH-2024-0003). All participants provided written informed consent prior to enrollment, in accordance with the Declaration of Helsinki. To ensure data anonymity, all personal identifiers (such as names and ID numbers) were removed and replaced with unique study codes (e.g., T-001). All research data were stored in a password-protected database, accessible only to the primary investigators.

### Research subjects

A total of 64 patients with T2DM admitted to the City People's Hospital were selected. To minimize confounding factors, a total of 36 male patients with T2DM were ultimately enrolled in this study after screening according to the predefined inclusion and exclusion criteria., with an average age of 52.78 ± 6.34 years, who were non-smokers and non-drinkers. Inclusion criteria were as follows: (1) All participants met the diagnostic criteria outlined in the 2013 edition of the ≪Chinese Guidelines for the Prevention and Treatment of T2DM≫; (2) No use of antibiotics or microecological preparations; (3) Diagnosed with T2DM for at least 2 years and not requiring insulin therapy; (4) Absence of diabetes-specific complications and ischemic heart disease, with the ability to engage in physical activity. Exclusion criteria included: (1) Female sex; (2) Endocrine disorders, inflammatory bowel diseases, or malabsorption syndromes; (3) Unhealthy lifestyle habits such as smoking, alcohol consumption, or substance abuse; (4) Use of antibiotics, probiotics, or medications affecting gastrointestinal function within the past month; (5) Regular exercise habits (moderate-intensity exercise >150 min per week); (6) Inability to comply with the Tai Chi intervention or follow-up procedures. All participants and their families provided written informed consent for the study.

### Research methods

#### Anthropometric measurements

Body composition was measured using the BCA-1B Body Composition Analyzer (Zhongti Tongfang Sports Technology Co., Ltd., China). After each startup and normal software debugging, the instrument was preheated for at least 30 min before measurement. Participants were instructed to remove outerwear and all metal items, stand barefoot with feet parallel on the foot electrodes, and grip the hand electrodes with arms naturally separated from the body at approximately 15°. They were asked to maintain an upright posture with chest out, head up, and eyes forward, remaining still throughout the test. Upon completion, the results were transmitted to a computer for data processing (13).

Height was measured using the DT02A Height and Weight Measuring Instrument. Participants stood barefoot, and during height measurement, the pressure plate was appropriately adjusted to contact the head while the observer ensured their line of sight was level with both the pressure plate and the scale (14).

Waist circumference was measured using a flexible tape measure, which was calibrated before use with a steel tape to ensure an error of no more than 0.2 cm per meter. Participants stood upright with arms slightly apart and hanging naturally, feet together, and body weight evenly distributed on both feet. The abdominal area was exposed, and participants were instructed to breathe calmly. During measurement, the tape was placed horizontally around the waist 0.5–1 cm above the upper edge of the umbilicus, and the measurement was taken at the end of exhalation. Results were recorded in centimeters, accurate to one decimal place (15).

### Collection and preservation of biological samples

Blood and stool samples were collected from all participants both before and after the training intervention. To ensure the accuracy of sampling results and minimize interference from other factors, all participants received detailed instructions one week prior to sample collection. These instructions were provided in two formats: a printed version distributed in person by the research team and an electronic version sent via WeChat. The specific reminders included the following:

- Avoid consuming yogurt, coffee, tea, energy drinks (e.g., Red Bull, Mizone), alcohol, and fermented foods such as pickles and fermented rice for three days prior to sampling.
- Refrain from eating spicy, greasy, or heavily processed foods, including organ meats, hot pot, skewers, marinated dishes, and fried foods.
- Do not use antibiotic medications (e.g., cephalosporins, amoxicillin, penicillin, roxithromycin) or other drugs such as traditional Chinese medicines, Chinese patent medicines, laxatives, or health supplements.
- Avoid engaging in strenuous exercise before sampling.
- Maintain a regular sleep schedule and avoid staying up late.

### Blood sample collection and preservation

Blood samples were collected from all participants. On the day of collection, participants arrived at the hospital laboratory's blood collection window between 7:00 and 9:00 a.m. after fasting for at least 10 h. Professional phlebotomists performed centralized blood draws. For each participant, three 5 ml tubes of fresh blood were collected from the cubital vein (primarily the median cubital vein). After collection, the blood samples were left to stand at room temperature for 30 min. Subsequently, they were centrifuged at 4 °C and 3,000 rpm for 5–10 min to separate the serum. Using a pipette, 1 ml of the supernatant was aliquoted into pre-labeled 1.5 ml sterile EP tubes. All aliquots were immediately stored in a −80 °C freezer.

### Biomarker assays

Serum biochemical parameters, including fasting insulin (FINS), fasting blood glucose (FBG), total cholesterol (TC), triglycerides (TG), low-density lipoprotein cholesterol (LDL-C), and high-density lipoprotein cholesterol (HDL-C), were measured using a Hitachi 7600 automatic biochemical analyzer. Specifically, glucose was analyzed by the hexokinase method, insulin by chemiluminescent immunoassay, and the four lipid parameters (total cholesterol, triglycerides, high-density lipoprotein cholesterol, and low-density lipoprotein cholesterol) by enzymatic colorimetric methods. Insulin resistance was evaluated using the Homeostasis Model Assessment of Insulin Resistance (HOMA-R) index. HOMA-R is a widely validated surrogate measure of fasting insulin resistance. It was calculated using the following standard formula: HOMA-R = (FBG × FINS)/405. A higher HOMA-R value indicates a lower level of insulin sensitivity and a higher degree of insulin resistance. All assays were performed strictly in accordance with the instrument operating procedures and reagent kit instructions, with quality control maintained using matched calibrators and controls (16).

Glycated hemoglobin (HbA1c) was measured using ion-exchange high-performance liquid chromatography (HPLC) with an ARKRAY HA-8180 automated glycohemoglobin analyzer. Original manufacturer-matched reagents and calibrators were employed for the analysis, and internal quality control procedures were implemented to ensure the validity of the results. The reference range for normal values was defined as HbA1c <7.0% (17).

Serum levels of TNF-α, IL-6, IL-1β, IL-10, CRP, D-LA, Zonulin, and MFG-E8 were measured using a K6600-A full-wavelength multifunctional microplate reader (Beijing, Kaiou) based on the double-antibody sandwich enzyme-linked immunosorbent assay (ELISA). Taking TNF-α as an example, the specific steps were as follows: (1) Coating: Microplate wells were pre-coated with TNF-α-specific capture antibodies. (2) Binding: TNF-α in serum samples was bound by the capture antibodies, and unbound substances were removed by washing. (3) Detection: Horseradish peroxidase (HRP)-labeled detection antibodies were added to form an “antibody-antigen-enzyme-labeled antibody” complex. (4) Color development: TMB substrate was added, and HRP catalyzed its color development, with the color intensity proportional to the TNF-α concentration. (5) Measurement: Absorbance was measured at 450 nm using the microplate reader, and TNF-α concentration was calculated based on a standard curve (18).

### Fecal sample collection and preservation

One day prior to fecal sample collection, participants were provided with individually sealed fecal collection kits and bio-ice packs. A plastic basin (with a diameter smaller than that of a household toilet bowl) was placed inside the toilet bowl, and a fresh-keeping bag was fitted over it (ensuring the edges of the bag did not come into contact with the toilet water). Participants defecated into the fresh-keeping bag. Using a long-handled spoon, the middle portion of the fresh stool was thoroughly mixed, and approximately 3–5 g of internal fecal matter from the morning stool was collected into the fecal collection container. A note indicating the sample collection time was prepared and placed together with the collected fecal sample and a pre-frozen ice pack. The sample was delivered to the testing laboratory within 2 h after collection and handed over to the research team. Upon receiving the specimen, the researchers immediately performed sample aliquoting on a dry ice box in the body fluid testing area. Each participant's fecal sample was divided into three pre-labeled 1.5 ml sterile EP tubes, with approximately 0.5 ml of sample per tube. The aliquots were then promptly stored in a −80 °C freezer.

### Gut microbiota sequencing

DNA was extracted using TIANGEN DNA extraction kits (TIANGEN Biotech, Beijing, China). The DNA concentration of each fecal sample was standardized to 50 ng/μl for sequencing. Amplification system construction: Specific primers containing barcode sequences were designed targeting the V3–V4 hypervariable region of the 16S rRNA gene, and a PCR amplification system was established using high-fidelity DNA polymerase. Product verification and recovery: Amplified products were verified by 2% agarose gel electrophoresis, and target DNA fragments were recovered by gel extraction. During the recovery process, precise quantification was performed using the Quant-iT PicoGreen dsDNA Quantitation Kit. Fluorescence quantification and library pooling: The recovered products were subjected to secondary quantification using a BioTek FLx800 microplate fluorescence reader. Based on the required sequencing depth, the quantified amplification products were pooled in equal proportions to construct the sequencing library.

Library preparation was completed using the Illumina TruSeq Nano DNA LT Library Prep Kit. Quality control was performed with an Agilent Bioanalyzer 2100, and library concentration was measured using Promega QuantiFluor. After confirming qualification, high-throughput sequencing was conducted on the Illumina platform.

Raw sequencing data (in FASTQ format) were first processed with cutadapt to remove primer sequences. DADA2 was then used for quality control steps, including quality filtering, denoising, merging, and chimera removal, ultimately generating a table of representative sequences and their abundance information. Species annotation of each representative sequence was performed using the QIIME 2 software package by aligning them with relevant databases. The 16S rRNA gene sequences were aligned against the Silva database (version 138), and taxonomic classification was conducted using the classify-sklearn tool with default parameters.

Alpha and beta diversity analyses were performed using QIIME 2. For alpha diversity analysis, the Shannon index (reflecting diversity) and Chao1 index (reflecting richness) were used to evaluate the richness and diversity of microbial communities within each group. Beta diversity analysis was conducted based on Bray-Curtis distance, and the significance of differences in gut microbial community structure between groups was further assessed using ANOSIM (Analysis of Similarities) (19).

### Intervention methods

Participants in our experimental group underwent a Tai Chi training intervention. Prior to enrollment, a safety assessment was conducted by a professional sports rehabilitation therapist as part of the inclusion/exclusion criteria. Based on participants‘ residential addresses and personal preferences, they were divided into four groups. Each group was led by a team leader (selected from the experimental group participants), who was responsible for recording attendance, organizing practice sessions, monitoring implementation, and documenting dietary conditions. For the first six weeks, a certified Tai Chi instructor provided detailed instruction and training for each movement to ensure all participants could perform the techniques correctly. Starting from the seventh week, each group leader organized practice sessions near participants' homes, with a frequency of five times per week. Each session consisted of 5–6 repetitions of the routine, lasting 30–40 min, and continued for six months. The Tai Chi routine used is the 24-Form Simplified Tai Chi promoted by the Chinese State General Administration of Sport; one complete practice takes about 6 min. Researchers will conduct unannounced and random inspections daily to ensure the study is completed with high quality (20).

#### Exercise intensity

The exercise intensity is primarily low-to-moderate, with heart rate = maximum heart rate × (50 %−60 %). Maximum heart rate = 220 – age. During exercise the heart rate must remain within a normal range known as the target heart rate. For aerobic exercise the reasonable heart-rate range = (maximum heart rate – resting heart rate) × 0.6 + resting heart rate to (maximum heart rate – resting heart rate) × 0.8 + resting heart rate (20).

#### Post-exercise subjective feelings

Heart rate noticeably increased, slight perspiration, and mildly labored breathing.

## Quality control

### Before the study

The experimental protocol was initially developed through group discussions, literature reviews, and suggestions from supervisors. Experts reviewed the feasibility and scientific nature of the protocol. Training and assessment were conducted for the research team members on Tai Chi, data collection, etc. Regular group discussions and analyses of the research progress were organized (21).

### Pilot study

A pilot study was conducted according to the initial protocol to identify and address any issues in a timely manner.

### Implementation phase

1) Each group leader organized the practice sessions and took attendance. Researchers conducted random inspections daily. Subjects with an attendance rate [attendance rate% = (number of training sessions attended/total planned training sessions) × 100%] of less than 70% were excluded. Free physical examinations were provided to those who completed the trial.
2) During the study, subjects were advised to engage in daily low-intensity activities such as walking and doing household chores but were not recommended to undertake additional high-intensity exercises. And, participants were instructed to refrain from initiating the use of any new medications, supplements, or functional foods known to significantly affect gut microbiota (e.g., probiotic drugs, lactic acid bacteria drinks) for the duration of the study. Those who were already on a stable regimen of such products prior to enrollment were allowed to continue their habitual intake unchanged, provided they documented and reported it. This protocol was designed to prevent the introduction of new confounding variables while maintaining ecological validity regarding participants' baseline practices.
3) Subjects were taught how to accurately record their daily activities, sleep conditions, and life and work status. Researchers collected these records on a regular basis.
4) Patients were instructed on how to recognize and manage symptoms of hypoglycemia and other discomforts during exercise to prevent adverse effects due to excessive intensity or prolonged duration of exercise.
5) During the study, participants were explicitly instructed to maintain their usual diet without modifications to their energy intake or dietary composition. Their dietary habits were monitored through structured 3-day dietary records at multiple time points, which were analyzed to confirm stability.

### Statistical analysis

Experimental data were analyzed using the SPSS 19.0 statistical software package, and GraphPad Prism 8 was used for graphing. Normally distributed measurement data were expressed as mean ± standard deviation (Mean ± SD). Non-normally distributed measurement data were presented as median and interquartile range. For result analysis, normally distributed measurement data were mainly analyzed using one-way analysis of variance (ANOVA) for statistical inference. Non-normally distributed measurement data were analyzed using the Wilcoxon Mann-Whitney U test. For correlation analysis, Pearson correlation was used for normally distributed data, while Spearman correlation was used for non-normally distributed data. A correlation coefficient less than 0.4 indicated a low correlation, 0.4–0.7 indicated a moderate correlation, and greater than 0.7 indicated a high correlation. A *p*-value of less than 0.05 was considered statistically significant.

## Results

### Anthropometric measurements and body composition

Compared with baseline, 6 months of Tai Chi training significantly reduced body weight, BMI, waist circumference, and body fat percentage (p <0.05) and significantly increased lean body mass (*p* < 0.05) in T2DM patients (Table 1).

**Table 1:** Comparison of anthropometric measurements and body composition before (T0) and after (T1) Tai Chi training.

**Parameters**	**T0 (*N* = 36)**	**T1 (*N* = 36)**	***t***	***p***
Weight (kg)	89.05 ± 15.17	76.29 ± 9.20^*^	2.28	0.035
Height (cm)	173.8 ± 9.52	175.9 ± 7.6	−0.58	0.591
BMI	29.47 ± 4.57	24.72 ± 3.11^*^	2.72	0.014
Waist (cm)	108.66 ± 8.33	100.02 ± 6.87^*^	2.53	0.021
Fat mass (%)	27.91 ± 2.74	24.11 ± 3.25^*^	2.84	0.011
Lean body mass (%)	70.37 ± 4.78	74.49 ± 3.64^*^	−2.17	0.044

Compared with T0, ^*^P < 0.05.

### Biochemical parameters

Compared with baseline, 6 months of Tai Chi training significantly decreased fasting blood glucose, HbA1c, HOMA-IR, CRP, TNF-α, IL-6, IL-1β, IL-8, and total cholesterol levels (*p* < 0.01) and significantly increased IL-10 levels (*p* < 0.01) in T2DM patients (Table 2).

**Table 2:** Comparison of blood glucose, serum inflammatory factors, and insulin resistance before (T0) and after (T1) Tai Chi training.

**Parameters**	**T0(*N* = 30)**	**T1(*N* = 30)**	***T***	***p***
Blood sugar (mg/dl)	146.26 ± 6.71	135.54 ± 11.23^*^	2.59	0.018
HbA1c (%)	7.93 ± 1.29	6.71 ± 0.69^*^	2.65	0.016
HOMA index	3.85 ± 0.57	3.05 ± 0.79^*^	2.55	0.021
CRP (mg/dL)	7.41 ± 1.18	4.85 ± 1.14^**^	4.95	<0.001
TNF-α (ng/mL)	2.35 ± 0.39	1.81 ± 0.29^**^	3.55	0.002
IL-6 (ng/mL)	7.45 ± 1.28	4.58 ± 0.6^**^	5.53	<0.001
IL-1β (pg/mL)	59.32 ± 7.74	47.06 ± 6.45^**^	3.85	0.001
IL-8 (μg/L)	25.62 ± 4.15	16.05 ± 2.62^**^	6.18	<0.001
IL-10 (ng/mL)	17.55 ± 4.38	25.44 ± 5.89^**^	−3.4	0.003
Triglyceride (mg/dL)	156.48 ± 12.72	133.16 ± 29.25^*^	2.41	0.027
Total cholesterol (mg/dL)	189.87 ± 42.74	138.79 ± 36.78^*^	2.87	0.01
LDL cholesterol (mg/dL)	89.98 ± 12.04	71.16 ± 13.27^**^	3.32	0.004
HD Lcholesterol (mg/dL)	56.55 ± 14.76	69.08 ± 15.88^*^	−2.49	0.023

HbA1c, Glycated hemoglobin; CRP, C-reactive protein HOMA Index Steady-state Model assessment index; LDL cholesterol, Low-density lipoprotein cholesterol; HDL cholesterol, High-density lipoprotein cholesterol; TNF-α, Tumor necrosis factor -α IL, Interleukin. Compared with T0, ^*^P < 0.05, ^**^P < 0.01.

### Gut microbiota analysis

**Figure 1:**
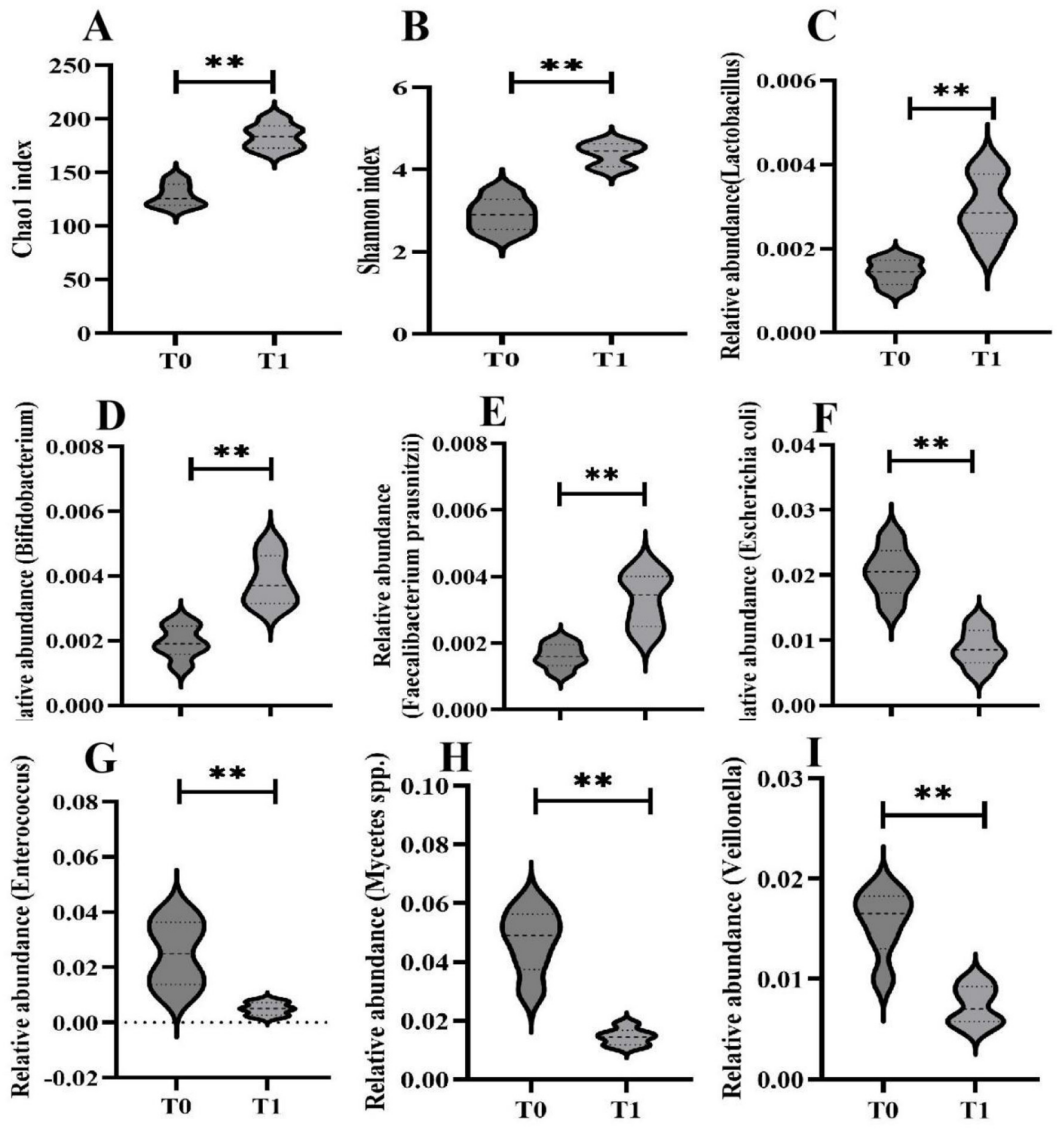
Comparison of gut microbiota before and after Tai Chi training. Compared with T0, **P < 0.01.

Compared with baseline, 6 months of Tai Chi training significantly increased the Chao1 and Shannon indices (*p* < 0.05) in T2DM patients. The abundance of *Lactobacillus, Bifidobacterium*, and *Faecalibacterium prausnitzii* increased significantly, while the abundance of *Escherichia, Enterococcus, Candida*, and *Veillonella* decreased significantly (*p* < 0.01) (Figures 1 A–H).

### Intestinal mucosal barrier function biomarkers

**Figure 2:**
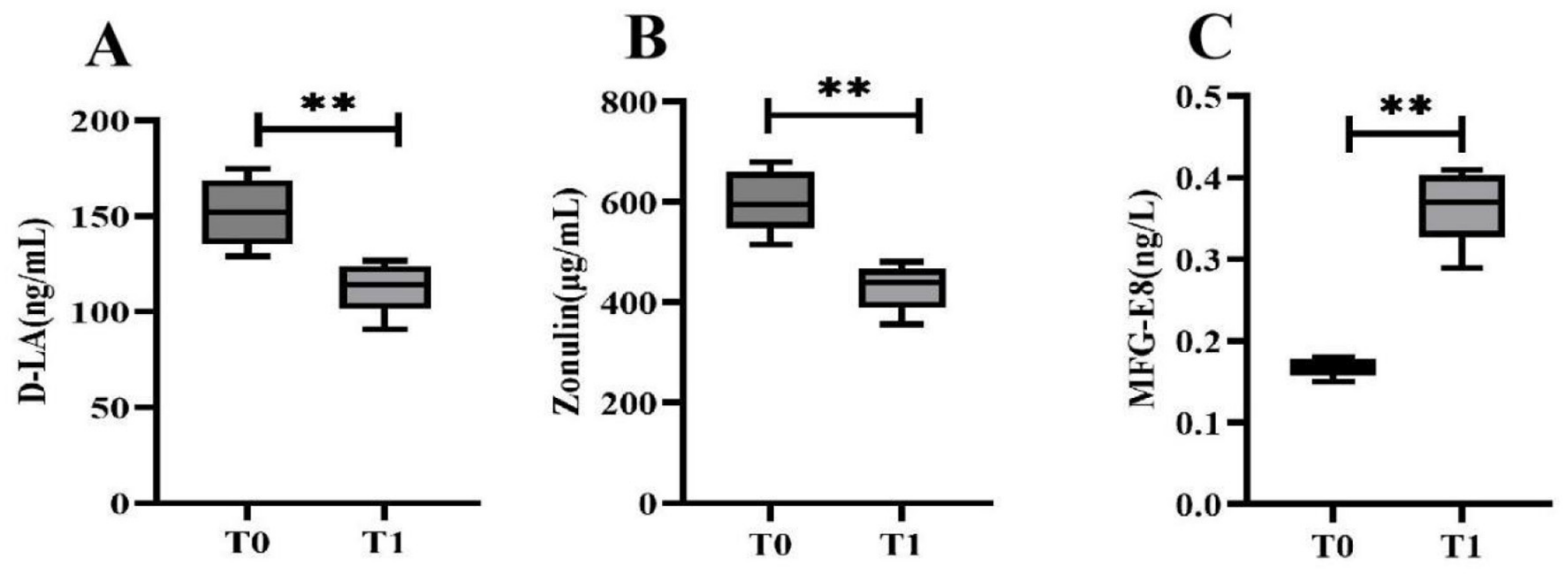
Comparison of intestinal mucosal barrier function biomarkers before and after Tai Chi training. D-lactate (D-LA), zonulin, and milk fat globule-EGF factor 8 (MFG-E8). Compared with T0, **P < 0.01.

Compared with baseline, 6 months of Tai Chi training significantly decreased the levels of intestinal mucosal barrier function biomarkers D-lactate and zonulin (*p* < 0.01) and increased the level of MFG-E8 (*p* < 0.01) in T2DM patients (Figures 2A–C).

### Correlation analysis results of gut microbiota, representative inflammatory molecules, and intestinal mucosal barrier function biomarkers

The results of correlation analysis showed that *Escherichia, Enterococcus, Candida*, and *Veillonella* were significantly positively correlated with the representative inflammatory molecule CRP and the intestinal mucosal barrier function biomarkers D-lactate and zonulin (*P* < 0.01), and significantly negatively correlated with MFG-E8 (*P* < 0.01). The representative inflammatory molecule CRP was significantly positively correlated with the intestinal mucosal barrier function biomarkers D-lactate and zonulin (*P* < 0.01), and significantly negatively correlated with MFG-E8 (*P* < 0.01) (Table 3).

**Table 3:** Correlations between gut microbiota, representative inflammatory molecules, and intestinal mucosal barrier function biomarkers.

**Parameters**	**D-LA**	**Zonulin**	**MFG-E8**	**CRP**
*Lactobacillus*	−0.062	−0.054	0.450^**^	−0.062
*Bifidobacterium*	−0.041	−0.015	0.398^**^	−0.183
*Faecalibacterium prausnitzii*	−0.050	−0.022	0.501^**^	−0.047
*Escherichia*	0.597^**^	0.687^**^	−0.545^*^	0.526^*^
*Enterococcus*	0.628^**^	0.691^**^	−0.582^*^	0.609^**^
*Mycetes* SPP.	0.791^**^	0.687^**^	−0.703^**^	0.693^**^
*Veillonella*	0.772^**^	0.712^**^	−0.588^**^	0.669^**^
CRP	0.589^**^	0.698^**^	−0.681^**^	

D-lactate (D-LA), zonulin, and milk fat globule-EGF factor 8 (MFG-E8). ^*^P < 0.05, ^**^P < 0.01.

## Discussion

The primary objective of this study was to investigate the regulatory effects of a 6-month Tai Chi training program on glucose homeostasis, serum inflammatory factors, and gut microbiota composition in patients with type 2 diabetes mellitus (T2DM). Our findings demonstrated that long-term Tai Chi intervention significantly improved body composition, characterized by a reduction in body fat percentage and a significant increase in lean body mass. Furthermore, the training led to enhanced glucose stability, as evidenced by significant decreases in fasting blood glucose, HbA1c, and the insulin resistance index (HOMA-IR). Regarding systemic inflammation, pro-inflammatory cytokines (such as CRP, TNF-α, and IL-6) were significantly downregulated, while the anti-inflammatory factor IL-10 was upregulated. Notably, Tai Chi training significantly increased the richness and diversity of the gut microbiota, promoting the abundance of beneficial bacteria (e.g., *Lactobacillus* and *Bifidobacterium*) while reducing opportunistic pathogens. Additionally, the significant improvement in intestinal barrier markers (decreased D-LA and Zonulin; increased MFG-E8) indicates that Tai Chi training effectively enhances gut mucosal integrity. These results suggest that the modulation of gut microbiota and the restoration of barrier function are key mechanisms through which Tai Chi exerts its therapeutic effects on T2DM.

In this study, we observed significant reductions in body weight, BMI, waist circumference, and body fat percentage, alongside a significant increase in lean body mass following Tai Chi training. The improvement in these anthropometric variables is of great clinical significance for T2DM patients. Obesity, particularly central obesity characterized by increased waist circumference, is a core driver of insulin resistance and metabolic dysfunction. As a moderate-intensity aerobic exercise, Tai Chi promotes fat oxidation and increases muscle mass through sustained energy expenditure and coordinated whole-body muscle engagement. The increase in lean body mass not only helps elevate the basal metabolic rate but also enhances glucose uptake and utilization by skeletal muscles, which largely explains the improvement in the insulin resistance index (HOMA-IR) observed in this study. Therefore, the optimization of body composition through Tai Chi serves as a fundamental mechanism for improving metabolic disorders in T2DM.

Studies have shown that changes in the structure and function of the gut microbiota are closely related to the T2DM phenotype, such as hyperglycemia and insulin resistance. The gut microbiota and its related metabolites play an important role in glucose metabolism, insulin resistance, and chronic inflammation. An imbalanced gut microbiota can weaken the intestinal mucosal barrier, thereby increasing systemic inflammatory responses, inducing insulin resistance, and promoting the development of T2DM (22). A large number of studies have confirmed that changes in the composition of the gut microbiota or ecological imbalance have been considered a potential environmental factor in the development of T2DM (23, 24). Studies have shown that in the gut microbiota of T2DM, butyrate-producing bacteria are reduced, while potential opportunistic pathogens increase (25). Combining animal and human studies, the overall differences in gut microbiota between T2DM patients and healthy controls are as follows: *Bifidobacterium, Bacteroides, Faecalibacterium, Akkermansia*, and *Roseburia* are negatively correlated with T2DM, while *Ruminococcus, Fusobacterium*, and *Blautia* are positively correlated with T2DM (26).

The structure and function of the gut microbiota in T2DM patients are different from those in healthy individuals, affecting the host's nutrition and energy metabolism and promoting fat synthesis and storage. Studies have shown that changes in the gut microbiota and different ratios of *Firmicutes to Clostridium, Lactobacillus*, and *Bacteroides* are associated with T2DM (27). Of course, these results are not comprehensive. The differences in gut microbiota changes observed in T2DM patients may be due to different races, dietary habits, lifestyles, environmental factors, and age and/or gender. In addition, the mechanisms causing ecological imbalance and the mechanistic links between gut microbiota changes and T2DM are not fully understood. The results of this study found that T2DM patients had reduced abundance and diversity of gut microbiota, increased abundance of pathogenic bacteria such as *Escherichia, Enterococcus, Candida, and Candida albicans*, and decreased abundance of beneficial bacteria such as *Lactobacillus* and *Bifidobacterium*. These data are consistent with previous results, indicating gut microbiota dysbiosis in T2DM patients. We speculate that gut microbiota dysbiosis may be a secondary cause of T2DM rather than its etiology. In fact, elevated blood glucose may create specific local conditions favorable for the colonization of intestinal pathogens, as studies have confirmed a correlation between blood glucose concentration and fungal colonization (28). Of course, fungi may also stimulate the systemic inflammation present in diabetes, which in turn leads to metabolic disorders, including poor glycemic control (29). Studies have shown that fungi interact with the innate immune receptor Dectin-1. Dectin-1 is a C-type lectin receptor that recognizes B-1,3-glucan present in most fungal cell walls. These receptors stimulate intracellular caspase signaling, leading to the production of pro-inflammatory cytokines and the activation of helper T lymphocytes (30). The results of this study also found that Escherichia, Enterococcus, Candida, and Candida albicans were significantly associated with elevated CRP. As is well known, low-grade inflammation is a cause of insulin resistance. In fact, inflammatory cytokines counteract the action of muscle insulin. For example, TNF-α induces insulin resistance by inactivating insulin receptor substrate-1 through receptor serine phosphorylation (31). In addition, activation of the innate immune system may lead to intestinal epithelial cell inflammation, changes in intestinal barrier function, and translocation of pathogens from the intestinal lumen to the patient's bloodstream, thereby amplifying the immune response (32). The results of this study cannot determine whether gut microbiota dysbiosis is a cause or a consequence of T2DM. However, gut microbiota dysbiosis may lead to the persistence and exacerbation of inflammation, which does affect T2DM. The results of this study also found that T2DM patients had significantly increased levels of D-lactate and zonulin and significantly decreased levels of MFG-E8. They are currently recognized serum markers of intestinal mucosal barrier function. Although the mechanisms of production of the three are different, they can all accurately reflect the state of intestinal mucosal barrier function (33). High concentrations of D-lactate and zonulin indicate a significant change in intestinal permeability, known as leaky gut (LG). This condition not only leads to impaired physiological function of the intestine but also allows gut microbes to translocate from the intestinal lumen to the circulatory blood, stimulating an immune response in our patients (34). Combining our research results, we found that the overgrowth of pathogens in the microbiota of T2DM patients is accompanied by a large increase in the content of D-lactate and zonulin, indicating the presence of intestinal barrier dysfunction and low-grade systemic inflammation affecting glucose metabolism, leading to intestinal fungal overgrowth, thereby forming a vicious circle.

As is well known, exercise can control blood glucose and inflammation, improve physical fitness, and change the body composition of T2DM patients (35). This study found that long-term Tai Chi training can improve glycemic homeostasis and insulin sensitivity in T2DM by reducing the abundance of intestinal pathogens, reducing systemic inflammation, and improving intestinal barrier function. However, it is not clear whether these improvements are due to improved diabetes control or occur independently. Studies in animals and some in humans have shown that exercise affects the gut immune function and microbiota of both T2DM patients and healthy subjects (36, 37). The mechanism by which exercise affects the gut microbiota is not fully understood. However, some data suggest that several factors can explain why exercise affects the gut microbiota. Animal experimental data indicate that exercise may alter the bile acid profile (7). It is well known that these compounds have some antimicrobial properties that affect the composition of the gut microbiota. These observations are also supported by significant changes in microbial characteristics caused by glycolate supplementation (38). Other experimental studies have shown that physical activity may alter fecal short-chain fatty acids (SCFAs), thereby increasing the presence of fecal butyrate and, in turn, increasing butyrate-producing gut bacteria (39). Studies have shown that SCFAs activate muscle AMPK, an enzyme that regulates muscle glucose and lipid metabolism. These metabolic effects may be important in diabetes and confirm the cross-talk between the microbiota, exercise, and overall metabolism (40). In addition, exercise may affect gut immune function (41). Animal studies have shown that long-term moderate physical activity increases the presence of intestinal immunoglobulin A (IgA) and reduces the impact of lymphocytes B and CD4+ T cells on cytokine gene expression, such as IL-6, IL-4, IL-10, and TGF-β, which are involved in IgA production. These modifications increase mucosal immunity, which can resist the colonization of intestinal pathogens (42). It is also meaningful to consider the possible indirect effects of exercise on gut microbiota and intestinal permeability. Previous data have shown that exercise stimulates the release of myokines from muscles, increases muscle glucose metabolism through AMPK activity, and reduces systemic inflammation (43). Systemic inflammation affects the metabolic homeostasis of intestinal epithelial cells, thereby affecting intestinal permeability and microbiota composition. Exercise also leads to weight loss. Human data show that the microbiota composition of obese and non-obese subjects is different, although it is not clear whether weight loss affects the microbiota or whether the microbiota leads to weight loss (44). The results of this study are consistent with previous observations, indicating that exercise is an effective strategy to improve the quality of life of T2DM patients. Specifically, 6 months of Tai Chi training significantly reduced the abundance of intestinal pathogens and systemic inflammation in T2DM, improved intestinal barrier function, and ultimately maintained glycemic homeostasis and insulin sensitivity. Treating the gut microbiota through physical exercise and/or specific therapies may be an important step toward customized therapy.

The findings of this study have substantial practical value. First, as a low-cost, low-risk, and easily disseminated traditional exercise, Tai Chi can serve as an important non-pharmacological adjunct for individuals with T2DM, helping to optimize individualized treatment plans and improve quality of life. Second, by highlighting the “gut microbiota–inflammation–metabolism” axis, our work provides a novel scientific rationale for exercise-based diabetes rehabilitation.

Nevertheless, several limitations should be noted. The study employed a self-controlled pre–post design without a parallel randomized control group, so we cannot fully rule out potential effects of time or other uncontrolled confounders. The sample size was relatively small (*N* = 36) and restricted to male participants, which may limit generalizability to broader populations (e.g., women or different age groups). Moreover, although we observed correlations between gut-microbiota shifts and metabolic indices, the specific molecular mechanisms through which Tai Chi modulates the microbiota remain to be elucidated.

Future research should address the following priorities: (i) include both sexes and enlarge sample size through multicenter trials to enhance external validity; (ii) extend follow-up duration to evaluate the long-term clinical benefits and persistence of Tai Chi interventions in T2DM; and (iii) integrate animal experiments or multi-omics approaches (e.g., metabolomics) to clarify the molecular pathways linking Tai Chi-induced microbiota changes to metabolic homeostasis.

## Conclusion

In the present study, patients with T2DM exhibited significant improvements in glycaemic control, systemic inflammatory status, and intestinal barrier function after a 6-month Tai Chi programme. These clinical gains were accompanied by marked increases in gut-microbial richness and diversity, together with favorable shifts in community composition. Although the absence of a parallel randomized control group precludes us from definitively attributing these changes solely to Tai Chi, the observed multidimensional biological improvements suggest that this mind–body exercise may represent a promising adjunctive strategy for personalized T2DM management. Large-scale, randomized controlled trials are warranted to confirm these findings and to establish a causal relationship between Tai Chi practice and enhanced metabolic health.

## Data Availability

The raw data supporting the conclusions of this article will be made available by the authors, without undue reservation. Anyone interested in using the data for scientific purposes is free to request permission from the corresponding authors. Email: chunyu3949@163.com.
